# Personal experiences in ultrasonography and sonoelastography of thyroid gland

**DOI:** 10.1186/1756-6614-8-S1-A22

**Published:** 2015-06-22

**Authors:** Marek Ruchala, Adam Stangierski, Kosma Woliński

**Affiliations:** 1 Department of Endocrinology, Metabolism and Internal Medicine; University of Medical Sciences; 49 Przybyszewskiego St, 60-355 Poznan, Poland

## 

In recent decades, thyroid ultrasonography has become one of the most important procedures performed in daily endocrinological practice. This convenient, fast, non-invasive and cheap procedure benefits in many aspects on determination of thyroid morphology.

The advantages of thyroid US include measurement of thyroid volume, evaluation of its echogenicity with visualization of parenchyma vascularisation. Also, thyroid ultrasonography is necessary for selection of so-called suspicious features of potential malignancy among thyroid lesions. According to numerous studies, combination of some specific sonographic features indicate higher risk of malignancy, and indicate necessity for fine needle aspiration biopsy (FNAB).

Sonographic features of the lesion, that rise the suspision of malignancy include: decreased echogenicity; irregular, diffused boarders; microcalcifications; local limphadenopathy; taller than wide orientation in parenchyma; increased vascular pattern in the center; documented, rapid growth of the lesion [1-3]. However, according to a recent meta-analysis by Brito et al., evaluating predictive values of different combination of those features, ultrasonography does not benefit in satisfactory values of sensitivity and specificity (table 1) [4].

**Table 1:** The main characteristics of studies included in the meta analysis.

Author	Year	Patients	Mean age	Nodules	Malignancies
Azizi et al. [6]	2012	706	women – 48.5, men – 47.7	912	86

Bojunga et al. [7]	2012	99 women, 39 men	52.0	158	21

Rossi et al. [8]	2012	1439 women, 417 men	52	2421	233

Trimboli et al. [9]	2012	438 women, 138 men	53.0	498	126

Bhatia et al. [10]	2011	89 patients*	not given	89	19

Merino et al. [11]	2011	89 women, 14 men	58	106	10

Ünlütürk et al. [12]	2011	157 women, 37 men	women - 43.7, men – 47.5	237	58

D’Souza et al. [13]	2010	151 women, 49 men	not given (range 8 – 74)	200	26

Friedrich-Rust et al. [14]	2010	37 women, 13 men	women – 54, men 52	53	7

Gietka – Czernel et al. [15]	2010	42 women, 10 men	45	71	22

Yunus et al. [16]	2010	58 women, 8 men	not given (range 18 – 75)	78	25

Asteria et al. [17]	2008	54 women, 12 men	women – 51.3, men – 60.5	86	17

Brunese et al. [18]	2008	264 women, 79 men	41.2	479	66

Rubaltelli et al. [19]	2008	25 women, 15 men	55	51	11

Similar results were obtained in recent meta-analysis performed in our center, in which newer publications had been evaluated and slightly different criteria for inclusion in the study had been used [5]. Only prospective studies were included. We have excluded studies focusing only on particular subgroups of patients and nodules – e.g. surgical or pediatric patients only, follicular lesions or lesions with previous non-diagnostic result of FNAB only etc*.* Finally, we analyzed the data of 5439 thyroid lesions. Just like in the paper of Brito, we have revealed the usefulness of some sonographic features in selecting potential malignancies. Both studies revealed significant value of “taller than wide feature” with positive predictive value of 76%. Different, independent features of higher risk of malignancy included hyopoechogenicity, and the presence of microcalcifications. According to both metaanalyzes, the value of Doppler analysis of the nodule vascularisation in prediction of thyroid malignancy, seems doubtful. (table 2, graph 1). We have also performed another meta-analysis dedicated specifically to sonographic features medullary thyroid cancers (MTCs); according to the pool results, MTCs presents similar sonographic appearance than other thyroid cancers (TCs); however, most markers of malignancy were less common for MTCs than papillary TCs (PTCs). Some features turned out to be important factors decreasing risk of MTC – e.g. none of the 157 included MTCs were hyperechogenic [6].

**Table 2:** Mean and median stiffness expressed in kPa in benign and malignant lesions. On the basis of: Szczepanek-Parulska E, Woliński K, Stangierski A, Gurgul E, Biczysko M, Majewski P, Rewaj-Łosyk M, Ruchała M. Comparison of diagnostic value of conventional ultrasonography and shear wave elastography in the prediction of thyroid lesions malignancy. *PLoS One*. 2013; **8(11)**: e81532.

	Mean	SD	median	P	range
**Q-box max [kPa]**					

Malignant	174.2	90.4	191.3	<0.0001	14.1-299.9
		
Benign	55.6	59.3	35.1		1.3-298.1

**Q-box mean [kPa]**					

Malignant	139.3	83.1	142.6	<0.0001	7.8-294.0
		
Benign	35.1	30.6	25.3		1.2-180.9

On the basis of: Woliński K, Szkudlarek M, Szczepanek-Parulska E, Ruchała M. Usefulness of different ultrasound features of malignancy in predicting the type of thyroid lesions: a meta-analysis of prospective studies. *Pol Arch Med Wewn*. 2014; **124**: 97-104.

**Figure 1:**
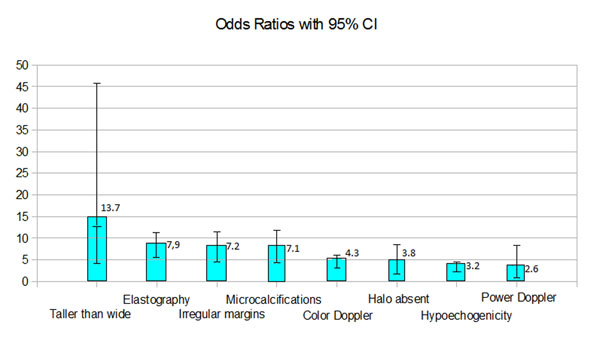
Pooled odds ratios with 95% confidence intervals for analysed sonographic markers of malignancy. On the basis of: Woliński K, Szkudlarek M, Szczepanek-Parulska E, Ruchała M. Usefulness of different ultrasound features of malignancy in predicting the type of thyroid lesions: a meta-analysis of prospective studies. *Pol Arch Med Wewn*. 2014; **124**: 97-104.

**Figure 2:**
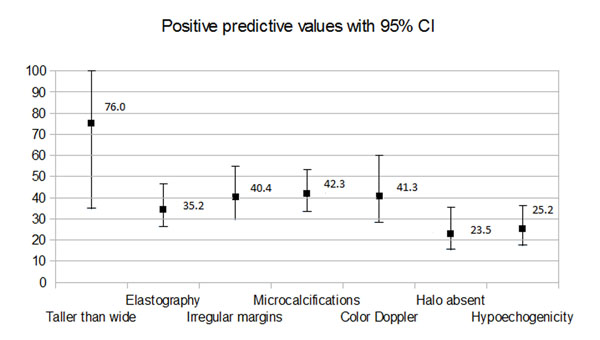
Pooled positive predictive values of significant sonographic markers of malignancy with 95% confidence intervals. On the basis of: Woliński K, Szkudlarek M, Szczepanek-Parulska E, Ruchała M. Usefulness of different ultrasound features of malignancy in predicting the type of thyroid lesions: a meta-analysis of prospective studies. *Pol Arch Med Wewn*. 2014; **124**: 97-104.

However, as the values sensitivity, specificity, PPV and NPV of thyroid ultrasonography as an independent procedure, are unsatisfactory, this procedure is not recommended for determination of thyroid malignancies and fine needle aspiration biopsy remains the “gold standard”. This invasive procedure, despite nowadays being the most accurate, it also has some disadvantages. Firstly, it’s invasive. Secondly, the whole diagnostic process, which includes ultrasonography, obtaining the material with the needle, fixation of the specimen and eventual cytological assessment by the pathologist is time consuming and quite expensive. Finally, there is a significant amount of indeterminate or inconclusive results (follicular lesions and smears inconclusive for adequate evaluation) [7]. Thus, exploration for new methods seems desirable.

Recently sonoelastography has been introduced in endocrinology practice, as an additional tool for ultrasonographic evaluation. This modern, non-invasive method, uses the acoustic radiation force in the assessment of the elasticity of examined tissue. Its value has been previously proven in the diagnosis of non-thyroidal oncologic conditions, such as breast cancer [8]. The first paper, describing its potential in thyroidology was published by Lyshchik et al. [9]. The researchers performed the procedures with the use of static, free-hand elastography, which demanded specific kind of compression, thus the results depended on the experience of the sonographist. Also, static elastography was time-consuming and did not provide adequate measurements of cystic and calcified lesions. As it did not let for quantitative measurement of the force needed for effective compression of the nodule, it was very subjective. Also, first generation of elastography was not reliable in selection of potential malignancies in multinodular goiter.

In 2010 Sebag et al., estimated the accuracy of shear wave elastography in non-invasive diagnosis of thyroid malignancies [10]. New method provided simultaneous quantitative and qualitative real time measurements without the need for manual compression of the tissue. Thusly, it was operator independent and highly repeatable. Sebag et al revealed very promising results, indicating high accuracy of SWE in the determination of thyroid malignancies. Recent years brought another information about potential use of SWE in monitoring the therapy of the subjects with acute, and subacute thyroiditis [11,12].

The potential of SWE in the diagnosis of thyroid malignancies had also been studied in detail in our center. The paper by Szczepanek-Parulska et al. included 122 patients with multinodular goiter (393 lesions) referred to our clinic prior total thyroidectomy [13]. Before the surgery each lesion was described in details, including the presence of suspected sonographic features and its elasticity. After the surgery, obtained specimens of each lesion was analyzed by an experienced pathologist. Basing on histopathological description, the study group included 18 papillary, two follicular, 1 medullary and 1 anaplastic thyroid carcinoma.

For the malignant lesions, the cut-off value of >50kPa was revealed as the most sensitive (OR 40.8, sensitivity 95%, specificity 70%). (table) Also SWE was found to be highly effective in the diagnosis of malignant lesions, with the use of quantitative color scale. Other sonographic markers of malignancy appeared to be significantly less accurate. (table 3)

**Table 3.** Usefulness of two qualitative scales (by Ueno and by Rago) in assessment of benign and malignant thyroid lesions. On the basis of: Szczepanek-Parulska E, Woliński K, Stangierski A, Gurgul E, Biczysko M, Majewski P et al. Comparison of diagnostic value of conventional ultrasonography and shear wave elastography in the prediction of thyroid lesions malignancy. *PLoS One*. 2013; **8(11)**: e81532.

Ueno scale	Malignant	Benign	OR	P
I	4.55%	45.74%	0.06 [0.008-0.424]	0.005

II	22.73%	38.76%	0.46 [0.17-1.29]	0.14

III	13.64%	12.92%	1.06 [0.30-3.73]	0.92

IV or V	59.09%	2.58%	58.1 [19.8-170.6]	<0.0001

**Rago scale**				

I	27.27%	84.50%	0.07 [0.03-0.18]	<0.0001

II	13.64%	12.92%	1.06 [0.30-3.73]	0.92

III	59.09%	2.58%	58.1 [19.8-170.6]	<0.0001

Another problem in endocrine practice is the issue of the selection of nodules for FNAB in case of multinodular goiter where the amount of lesions can be high and it is not possible to puncture all nodules. Study performed in our department showed that SWE is also valuable tool in the selection of lesions for FNAB [14]. All analyzed cancers turned out to be the least elastic lesions in particular goiters; even ones which were not very stiff in absolute values were stiffer than other lesions present in the same goiter.

Despite many benefits and high diagnostic value in differentiation of benign and malignant thyroid lesions SWE is not free of limitations. Some features were described as interfering results of sonoelastographic examination and potentially leading to overestimation of the cancer risk. According to the study performed by Bhatia et al. [15] partially cystic lesions were less elastic than solid ones; stiffness was also positively correlated with the diameter of the nodule. Study performed in our department brought the first, systematic analysis of biochemical and ultrasonographic parameters influencing elasticity of thyroid nodules [16]. According to our results, numerous parameters can increase stiffness of the lesion. Most important among them were micro- and especially macrocalcifications, cystic components, isthmal location; stiffness was also correlated with the maximal diameter of the lesion.

In conclusion, conventional sonographic markers of malignancy seem to be valuable for the preliminary assessment of thyroid nodules; however, these features do not benefit in satisfactory values of sensitivity and specificity. Elastography and particularly SWE seems to be important advance of conventional ultrasonography allowing for the more reliable distinction between benign and malignant thyroid nodules as well as better selection of lesions for FNAB in case of multinodular goiter. However, SWE can be not credible in case of some lesions (e.g. partially cystic, with calcifications, *etc.*). Also data about usefulness of SWE in case of some particular types of thyroid cancer – such as follicular and medullary TCs are very limited. Altogether, there is still a need for further techniques allowing for more reliable distinction between benign and malignant thyroid nodules as well as further studies on the available techniques.

## References

[B1] Rossi M Buratto M Bruni S Filieri C Tagliati F Trasforini G Role of Ultrasonographic/Clinical Profile, Cytology, and BRAF V600E Mutation Evaluation in Thyroid Nodule Screening for Malignancy: A Prospective Study J Clin Endocrinol Metab 2012 97 7 2354 2361 10.1210/jc.2011-3494 22535974

[B2] Gietka-Czernel M Kochman M Bujalska K Stachlewska-Nasfeter E Zgliczyński W Real-time ultrasound elastography - a new tool for diagnosing thyroid nodules Endokrynol Pol 2010 61 6 652 657 21104638

[B3] Frates MC Benson CB Charboneau JW Cibas ES Clark OH Coleman BG Management of thyroid nodules detected at US: Society of Radiologists in Ultrasound consensus conference statement Radiology 2005 237 3 794 800 10.1148/radiol.2373050220 16304103

[B4] Brito JP Gionfriddo MR Al Nofal A Boehmer KR Leppin AL Reading C The accuracy of thyroid nodule ultrasound to predict thyroid cancer: systematic review and meta-analysis J Clin Endocrinol Metab 2014 99 4 1253 1263 10.1210/jc.2013-2928 24276450 PMC3973781

[B5] Woliński K Szkudlarek M Szczepanek-Parulska E Ruchała M Usefulness of different ultrasound features of malignancy in predicting the type of thyroid lesions: a meta-analysis of prospective studies Pol Arch Med Wewn 2014 124 3 97 104 24473342 10.20452/pamw.2132

[B6] Woliński K Rewaj-Łosyk M Ruchała M Sonographic features of medullary thyroid carcinomas--a systematic review and meta-analysis Endokrynol Pol 2014 65 4 314 18 10.5603/EP.2014.0043 25185855

[B7] Stangierski A Wolinski K Martin K Leitgeber O Ruchala M Core needle biopsy of thyroid nodules - evaluation of diagnostic utility and pain experience Neuro Endocrinol Lett 2013 34 8 798 801 24522016

[B8] Ueno E Ito A Diagnosis of breast cancer by elasticity imaging Eizo Joho Medical 2004 36 12 2 6

[B9] Lyshchik A Higashi T Asato R Tanaka S Ito J Mai JJ Thyroid gland tumor diagnosis at US elastography Radiology 2005 237 1 202 211 10.1148/radiol.2363041248 16118150

[B10] Sebag F Vaillant-Lombard J Berbis J Griset V Henry JF Petit P Oliver C Shear wave elastography: a new ultrasound imaging mode for the differential diagnosis of benign and malignant thyroid nodules J Clin Endocrinol Metab 2010 95 12 5281 5288 10.1210/jc.2010-0766 20881263

[B11] Sporea I Sirli R Bota S Vlad M Popescu A Zosin I ARFI elastography for the evaluation of diffuse thyroid gland pathology: Preliminary results World J Radiol 2012 4 4 174 178 10.4329/wjr.v4.i4.174 22590672 PMC3351686

[B12] Ruchala M Szczepanek-Parulska E Zybek A Moczko J Czarnywojtek A Kaminski G Sowinski J The role of sonoelastography in acute, subacute and chronic thyroiditis: a novel application of the method Eur J Endocrinol 2012 166 3 425 432 10.1530/EJE-11-0736 22143319

[B13] Szczepanek-Parulska E Woliński K Stangierski A Gurgul E Biczysko M Majewski P Rewaj-Łosyk M Ruchała M Comparison of Diagnostic Value of Conventional Ultrasonography and Shear Wave Elastography in the Prediction of Thyroid Lesions Malignancy PLoS One 2013 8 11 e81532 10.1371/journal.pone.0081532 24312313 PMC3843667

[B14] Woliński K Szczepanek-Parulska E Stangierski A Gurgul E Rewaj-Łosyk M Ruchała M How to select nodules for fine-needle aspiration biopsy in multinodular goitre. Role of conventional ultrasonography and shear wave elastography - a preliminary study Endokrynol Pol 2014 65 2 114 118 10.5603/EP.2014.0016 24802734

[B15] Bhatia KS Rasalkar DP Lee YP Wong KT King AD Yuen HY Ahuja AT Cystic change in thyroid nodules: a confounding factor for real-time qualitative thyroid ultrasound elastography Clin Radiol 2011 66 9 799 807 10.1016/j.crad.2011.03.011 21530955

[B16] Szczepanek-Parulska E Woliński K Stangierski A Gurgul E Ruchała M Biochemical and ultrasonographic parameters influencing thyroid nodules elasticity Endocrine 2014 47 2 519 527 10.1007/s12020-014-0197-y 24535467

